# Clinical Determinants of Childhood Onset Systemic Lupus Erythematosus among Early and Peri-Adolescent Age Groups

**DOI:** 10.3390/children9121865

**Published:** 2022-11-30

**Authors:** Meghan Corrigan Nelson, Shanmuganathan Chandrakasan, Lori Ponder, Ignacio Sanz, Baruch Goldberg, Ekemini A. Ogbu, Kelly Rouster-Stevens, Sampath Prahalad

**Affiliations:** 1 Department of Pediatrics, School of Medicine, Emory University, Atlanta, GA 30307, USA; 2 Children’s Healthcare of Atlanta, Atlanta, GA 30307, USA; 3 Aflac Cancer and Blood Disorders Center, Children’s Healthcare of Atlanta, Emory University, Atlanta, GA 30307, USA; 4 Department of Medicine, Division of Rheumatology, Lowance Center for Human Immunology, Emory University, Atlanta, GA 30307, USA; 5 Division of Rheumatology, Cincinnati Children’s Hospital Medical Center, Cincinnati, OH 45229, USA; 6 Department of Pediatrics, University of Cincinnati College of Medicine, Cincinnati, OH 45229, USA; 7 Department of Pediatrics, Johns Hopkins University, Baltimore, MD 21218, USA; 8 Department of Human Genetics, School of Medicine, Emory University, Atlanta, GA 30307, USA

**Keywords:** childhood onset SLE, lupus nephritis, early-onset cSLE, peri-adolescent onset cSLE

## Abstract

Introduction: Systemic lupus erythematosus (SLE) is a multisystem autoimmune disease that is associated with significant morbidity and mortality. SLE disproportionately affects women and minorities. Childhood-onset SLE (cSLE) in particular tends to be more aggressive than adult-onset SLE. Despite substantial improvements in the treatment of cSLE, there is significant variability in treatment responses and long-term outcomes. Furthermore, there is a paucity of studies involving cSLE, and in particular, cSLE among different age groups. The aim of this study was to test the hypothesis that an early-onset cSLE cohort would demonstrate unique characteristics with distinctive clinical and laboratory features at disease onset. We specifically investigated whether clinical, epidemiological, or serological factors are differentially associated with early- and late-onset cSLE. This could have direct impact on clinical management with the goal of improving outcomes and quality of life for children with SLE. Methods: Our study was conducted at a large tertiary center. We included 213 subjects seen at our pediatric rheumatology clinic aged 4–17 years. Epidemiologic, clinical phenotype, disease severity, serology, treatment, and outcome data were compared between subjects with cSLE onset prior to 10 years of age (early-onset disease, *n* = 43) and those with cSLE onset greater than 10 years of age (peri-adolescent disease, *n* = 170). We compared clinical features between early- and peri-adolescent onset cSLE in order to investigate the association between age at disease onset of cSLE and clinical disease expression and outcomes. Results: Of the 213 subjects with cSLE in our study, 43 subjects had early-onset disease (age 2 to ≤9 years) and 170 patients had peri-adolescent onset disease. We found that early-onset cSLE was associated with a higher prevalence of positive anti-dsDNA antibody at cSLE diagnosis, higher anti-dsDNA antibody titer at cSLE diagnosis, rash, and azathioprine use (*p* < 0.001, *p* = 0.004, *p* = 0.011, and *p* = 0.008, respectively). In contrast, we found that peri-adolescent onset cSLE (≥10 years of age) was associated with worse disease activity (SLEDAI range 0–24) (*p* < 0.001), higher SLICC at diagnosis (*p* < 0.001), as well as a higher rate of mycophenolate mofetil and hydroxychloroquine use (*p* = 0.003 and *p* < 0.001, respectively). There were no significant differences in the prevalence of neuropsychiatric symptoms or the development of Class IV/Class V lupus nephritis between the early-onset and peri-adolescent groups.

## 1. Introduction

Systemic lupus erythematosus (SLE) is a multisystem autoimmune disease that is associated with significant organ damage, morbidity, and mortality [1,2,3,4]. Approximately 15–20% of SLE patients develop the disease before their 18th birthday and can be considered to have childhood-onset SLE (cSLE). Despite substantial improvements in the treatment of cSLE, there remains significant disease-related morbidity [1,5]. cSLE is associated with more severe organ involvement, including renal and CNS disease, increased disease activity, the presence of greater damage at the time of diagnosis, and a higher steroid burden, contributing to the increased morbidity and mortality when compared with adult-onset [1,6]. In contrast with adult-onset SLE, cSLE has been associated with more frequent use of corticosteroids and other immunosuppressive agents reflective of increased disease activity [1,7].

Despite the reported increased severity of cSLE compared to adults, there have been conflicting data regarding outcomes amongst the different pediatric age groups [5]. Additionally, there is a paucity of data pertaining to disease characteristics as well as therapies utilized to induce remission in pre-pubertal versus adolescent-onset cSLE [5,7,8,9,10]. Previous literature have reported a similar disease course between early-onset (defined as 0–9 years) and later onset (defined as 10–19 years) disease [10,11]. In previously published reports, there are similarities in the prevalence of mucocutaneous, gastrointestinal, and lymphadenopathy amongst different age groups in cSLE [5,10]. While there are conflicting reports of associated hypocomplementemia amongst different age groups [5,8,9], previous studies have not found a significant difference between pre-pubertal versus adolescent-onset cSLE and disease activity, as represented by the Systemic Lupus Erythematosus Disease Activity Index (SLEDAI) [8,10]. Previous literature have supported that overall disease severity, in particular, cytopenias, as well as cardiac, renal, and pulmonary involvement, are inversely related with the age at onset; this finding was emphasized with early-onset SLE [5,9]. Past studies have found that musculoskeletal involvement, including arthritis, increases with the age of onset [5,9]. It has been proposed that adolescent-onset cSLE patients have a strong female predilection, while early-onset cSLE has no gender predilection [5]. With respect to medication utilization, previous literature have supported that early-onset cSLE typically requires increased dosages of corticosteroids as well as an overall increased number of immunosuppressive medications to achieve remission [10]. Prior studies evaluating cSLE amongst age groups have not determined any significant differences with respect to rituximab, cyclophosphamide, mycophenolate, hydroxychloroquine, or azathioprine use at the time of enrollment [10].

The aim of this study was to investigate the association of age at disease onset on disease progression and outcomes of childhood systemic lupus erythematosus (cSLE), to include the development of Class IV/V lupus nephritis, neuropsychiatric disease, and disease activity prior to adulthood. We conducted this study to analyze the clinical features of patients with cSLE with onset stratified by age. We elucidated that the early-onset cSLE cohort demonstrates unique characteristics with distinctive clinical features in contrast to the peri-adolescent onset cSLE group, as well as anticipate a correlation with increased disease severity and morbidity. This information would be beneficial in predicting outcomes and guiding the clinical management of children with cSLE.

## 2. Methods

We included patients newly diagnosed with cSLE between January 2000 and July 2021, and followed at the Pediatric Rheumatology Clinics at Emory University and Children’s Healthcare of Atlanta (CHOA). These patients were enrolled in the South Eastern Registry for Childhood Arthritis Study (SEARCH) and/or Immunological Characterization of Pre-Clinical Autoimmunity in Pediatric Patients Study (PACMAN). Only subjects that had at least one year of follow up were included. Our studies were approved by the Institutional Review Board at Emory University in Atlanta, GA, USA, and all study participants gave informed consent. Forty-three patients diagnosed with cSLE prior to their tenth birthday were included in the “early onset cSLE cohort.” One hundred seventy patients diagnosed between their tenth and eighteenth birthdays were included in the “peri-adolescent onset cSLE cohort.” Ten years of age was defined as the cut off between groups in order to capture patients prior to the average age of puberty, allow sufficient sample sizes, as well as reflect the median age of onset of cSLE (ages 11–12) [12,13].

All patients met at least four of the 17 Systemic Lupus International Collaborating Clinics (SLICC) classification criteria for SLE. For the SLICC criteria, this included at least one clinical and one immunologic criterion [14,15]. Demographic, laboratory results, and clinical data from the time of diagnosis to July 2021 were extracted from the Electronic Medical Record (EMR) utilized at CHOA. SLE disease activity was measured using the SLE Disease Activity Index (SLEDAI), utilizing documented EMR data [16]. Disease duration was defined as the duration from the time of onset to the time of enrollment. Patients with renaldysfunction, as evidenced by abnormal renal function, persistent proteinuria, or elevated creatinine, underwent renal biopsy and the pathology was classified according to the International Society of Nephrology classification for lupus nephritis [14,15]. Patients diagnosed with central nervous system (CNS) disease met the criteria for one or greater neuropsychiatric syndromes in SLE [17].

Subject data were obtained at the time of enrollment. Descriptive statistics as well as Wilcox Rank Sum and Spearman’s rank correlation coefficients were performed with the data obtained. Additionally, a false discovery rate (FDR) correction was performed to account for multiple comparisons by the Benjamin–Hochberg procedure (corrected *p*-value). The *p*-value for Spearman’s rank and Wilcox Rank sum correlation coefficient as well as FDR correction was calculated using the SPSS Statistics Data Editor (v28.0.1.1 [7] (IBM Corp: Armonk, NY, USA) with a *p*-value of ≤0.05 indicating significance.

## 3. Results

*Demographics*: Within our cohort, there was an increased frequency of Black females, with a mean age of 12.1 ± 3.2 years of age (Table 1). In contrast, within our patient population, only 20% were males. Within our early-onset cohort, the age of onset was a mean of 7.0 ± 1.5 years of age in contrast to our peri-adolescent cohort, with the mean age of 13.2 ± 2.1 (Table 1).

*SLE related clinical characteristics*: At the time of our study, disease duration was a mean of 3.0 ± 3.0 years (Table 1). Overall, early-onset cSLE had an increased disease duration of a mean average of 5.0 ± 3.23 years, in contrast to older onset cSLE with 3.0 ± 2.1 years at the time of enrollment (Table 1). The mean SLEDAI score was 6.1 ± 5.0. Approximately 20.7% of patients had Class IV/V lupus nephritis, and 5.2% reported neuropsychiatric lupus symptoms (Supplemental Table S1). Approximately half of patients at diagnosis presented with cytopenias and 76% with rash. Arthritis was more common among the peri-adolescent onset cSLE cohort compared to the early-onset cSLE cohort (42.9% versus 37%, respectively; *p* = 0.04 with FDR corrected *p*-value *p* = 0.09), whereas the early-onset cSLE cohort had an increased prevalence of rash (93% vs. 72.4%, respectively; *p* = 0.011).

With respect to disease severity, at the time of diagnosis our cohort had an average SLEDAI score of 9.75 ± 4.68 (Table 1) in comparison with prior studies, which report a mean SLEDAI of 4.8 ± 5.6 among their cSLE cohort [18]. Of interest, our peri-adolescent onset cSLE group had a higher mean SLEDAI score of 9.73 ± 4.90 in contrast to our early-onset cSLE of 9.68 ± 3.9 (*p* < 0.001). Furthermore, our peri-adolescent onset cSLE group met a greater number of SLICC criteria at diagnosis (5.3 ± 1.9) compared to the early-onset cSLE group (4.9 ± 1.6); (*p* < 0.001).

*Biomarkers at diagnosis:* At the time of diagnosis, there were a similar proportion of anti-Smith antibodies in both our early and peri-adolescent onset cohorts (Table 1). In the early-onset cSLE cohort, there was a higher frequency of anti-dsDNA antibodies as well as a high titer (1:640 and higher) of anti-dsDNA antibodies (*p* < 0.001, *p* = 0.004, respectively). The early-onset cSLE cohort had a lower prevalence of hypocomplementemia and a higher prevalence of cytopenias at diagnosis compared to the peri-adolescent onset cSLE cohort, although this difference was not significant (Table 1).

*Medications utilized*: Comparing age groups, we found that 11.1% and 9.4% of the peri-adolescent onset cSLE group had received cyclophosphamide and rituximab at the time of enrollment, respectively (Table 2), although this difference was not significant. A significantly greater proportion of the peri-adolescent onset cSLE group were taking mycophenolate (42.3%; *p* = 0.003) and hydroxychloroquine (84.7%; *p* < 0.001). By contrast, the early-onset cSLE group was on azathioprine more frequently (12%; *p* = 0.008). In all, 71% of our cohort were taking prednisone at the time of enrollment, with 42% of those requiring high dose prednisone (defined as ≥15 mg daily). When our cohorts were compared, approximately 45.2% of the peri-adolescent onset cSLE group were on high dose prednisone in contrast to 27.9% of the early-onset cSLE at enrollment (Table 2); this difference was not statistically significant.

## 4. Discussion

This study sought to identify the unique clinical characteristics that differentiate children with younger (<10 years) versus older (10–18 years) age at cSLE onset. In particular, we have found that overall, the peri-adolescent onset cSLE group (≥10 years of age) was associated with worse disease activity (SLEDAI) and a higher frequency of arthritis development at diagnosis despite lesser disease duration. Interestingly, there were no significant differences between the age groups with respect to predilection towards neuropsychiatric disease or development of Class IV/Class V glomerulonephritis. These findings suggest that disease activity may be more severe in the peri-adolescent onset group at diagnosis in part due to the extensive number of organ involvement as per SLICC criteria, while disease severity in the early-onset cohort may increase over time, a finding previously supported by Massias et al. [8].

Our clinic is located at an urban tertiary care center; with respect to demographics, our patient population had a higher predominance of Black females in comparison with prior cohorts [18]. As non-White race has been associated with greater disease activity and end organ dysfunction in SLE, the disease burden observed was not unforeseen [19,20,21,22]. With respect to medication utilization, previous studies have also shown that a similar proportion of patients were requiring prednisone use across age groups, as with our patient cohort [10]. Our patients had an overall lower frequency of use of cyclophosphamide and higher frequency of use of mycophenolate and rituximab in contrast to previous literature [18]. Of note, of the patients who utilized azathioprine at the time of enrollment, a majority of these patients were diagnosed with autoimmune-mediated cytopenias and two were diagnosed with autoimmune hepatitis prior to initiation of this therapy. Approximately 40% of patients treated with azathioprine had previously received cyclophosphamide or rituximab prior to the initiation of azathioprine. Azathioprine was specifically more commonly used with cSLE diagnoses in 2015 and earlier, and therefore, may also reflect the trends of clinical practice at that time.

Amongst our cSLE patients, while there were similarities noted with respect to anti-Smith antibodies and serositis at diagnosis, we did note that there was an increased frequency of anti-dsDNA antibodies at diagnosis as well as a high titer of anti-dsDNA antibody (1:640 or higher) for the early-onset cSLE cohort. This is in partial corroboration with previous studies, which have reported higher anti-dsDNA titers and less frequency of hypocomplementemia in prepubertal cSLE patients [9]. Overall, these differences are suggestive of variable pathomechanisms for disease development across age groups.

Of note, previous studies have shown that genetics play a vital role in susceptibility to developing SLE [23]. It has been postulated that, given the earlier onset of cSLE as well as increased disease severity, there may be a higher genetic burden of disease onset with cSLE when compared with adult-onset SLE. Additionally, it has been proposed that rare monogenic forms of SLE are genetically enriched in the cSLE patient population, specifically to include abnormalities of the early complement pathways as well as genes encoded in sensing DNA and RNA [23]. Our findings overall suggest there may be variability with respect to the pathomechanisms responsible for driving disease development in our younger onset. Further studies are needed to determine if the early-onset cSLE cohort is genetically enriched and if so, which pathways are enhanced.

As there is a paucity of information pertaining to the clinical expression between the age groups of childhood-onset SLE, the strength of this study is in directly expanding our limited knowledge pertaining to the clinical data in cSLE within the scientific community. Additionally, this is one of the largest studies to date within the United States comparing the differences among childhood-onset lupus. Given the limited number of studies specifically comparing clinical distinctions between the subgroups of childhood-onset SLE, this study will directly contribute to our lack of knowledge in this field [5,9,10].

Medication non-adherence and socioeconomic status, previously shown to be associated with worse disease severity, were not specifically evaluated in our study, but may have contributed to the disease burden observed [24,25,26]. Additional limitations of this study include the reliance upon the electronic medical record for chart review, which directly impacted SLEDAI assessment as well as reported clinical and laboratory related features. Given this, reliance upon the EMR may present an element of reporting bias. Additionally, given that cSLE is a rare disease, further studies are needed in order to fully elucidate this association. Increased sample sizes would also provide for further differentiation of identified trends within other ethnically diverse patient populations, as our cohort was predominantly Black females. Although our cohort was large, data were obtained at a single institution where disease burden is significant with an overall age-adjusted prevalence rate among the highest reported in the United States and therefore, our results may be impacted by disease prevalence and relative severity; therefore, future multi-institutional studies would be representative of a more heterogenous patient population, such as by using larger banked registries [24]. Future studies should also assess disease activity as well as investigate the role of puberty, as per the Tanner Stages, in disease development, medications utilized for disease control, and outcome [27,28,29]. Further studies should continue to look at long-term outcomes and longitudinal data as a means to assess overall disease outcome in this particularly vulnerable patient population.

## 5. Conclusions

This is one of the largest cohorts in the United States to date comparing clinical and laboratory features of cSLE patients between age groups (onset <10 and ≥10 years of age). Our study has shown that there are distinct clinical differences between the two age groups. Interestingly, we did not find a difference in the prevalence of neuropsychiatric disease or Class IV/V lupus nephritis between the age groups. Consistent with previous studies, disease activity overall is higher in older cSLE patients, although the pathomechanism of this remains unclear.

## Figures and Tables

**Table 1:** Demographics and Clinical Features of Enrolled Subjects with Childhood-onset Systemic Lupus Erythematosus Stratified by Age ^1^.

Demographics	Our Cohort (*n* = 213)	Early-Onset cSLE [Age at Onset *<*10] (*n* = 43)	Peri-Adolescent Onset cSLE [Age at onset ≥10] (*n* = 170)	*p*-Value	Corrected *p*-Value
**Gender**				0.112	0.248
Female	*170 (79.8%)*	*33 (77%)*	*137 (80.5%)*		
Male	*43 (20.2%)*	*10 (23%)*	*33 (19.4%)*		
Age of onset, mean ± SD (median, range)	12.1 ± 3.2 (13.0, 4–17)	7.0 ± 1.5 (7.0, 4–9)	13.2 ± 2.1 (14.0, 10–17)		
**Race**				**<0.001**	**<0.001**
White	*59 (27.6%)*	*17 (39.5%)*	*42 (24.7%)*		
Black	*136 (63.8%)*	*25 (58.1%)*	*112 (65.8%)*		
Asian	*14 (6.6%)*	*1 (2.3%)*	*14 (8.2%)*		
Other	*4 (1.9%)*	*0*	*4 (2.3%)*		
**Disease Characteristics**					
SLE Disease duration in years, mean ± SD (median, range)	3.0 ± 3.0 (3.0, 0–13)	5.0 ± 3.23 (4.0, 0–13)	3.0 ± 2.1 (2.0, 0–10)	**<0.001**	**<0.001**
SLEDAI-2K, mean ± SD (median, range) at diagnosis	9.75 ± 4.68 (10.0, 1–32)	9.68 ± 3.9 (10.0, 2–17)	9.73 ± 4.90 (9.0, 1–32)	**<0.001**	**<0.001**
SLEDAI-2K, mean ± SD (median, range) at enrollment	6.1 ± 5.0 (5.0, 0–24)	5.6 ± 4.9 (4.0, 0–17)	6.26 ± 5.00 (5.0, 0–24)	**<0.001**	**<0.001**
SLICC Criteria, mean ± SD (median, range)	5.2 ± 1.8 (5.0, 1–12)	4.9 ± 1.6 (5.0, 1–9)	5.3 ± 1.9 (5.0, 1–12)	**<0.001**	**<0.001**
SLEDAI, ≥10	*44 (20.7%)*	*9 (20.9.%)*	*35 (20.5%)*	0.7	1.0
SLICC Disease Index	0.63 ± 0.62 (1.0, 0–2.0)	0.56 ± 0.60 (1.0, 0–2.0)	0.65 ± 0.63 (1.0, 0–2.0)	0.876	1.0
Class II/III lupus nephritis	*5 (2.3%)*	*0*	*5 (2.9%)*	1.0	1.0
Class IV/V lupus nephritis	*44 (20.7%)*	*9 (20.9%)*	*35 (20.5%)*	0.7	1.0
Neuro-psychiatric	*11 (5.2%)*	1 *(2.3%)*	*10 (5.8%)*	0.579	1.0
Arthritis	*89 (41.8%)*	*16 (37%)*	*73 (42.9%)*	**0.046**	**0.09**
Serositis	*43 (20.2%)*	*7 (16%)*	*33 (19.4%)*	0.936	1.0
Cytopenia	*118 (55.4%)*	*26 (60.4%)*	*92 (54.1%)*	0.533	0.710
Rash	*163 (76.5%)*	*40 (93.0%)*	*123 (72.4%)*	**0.011**	**0.029**
**Biomarkers at diagnosis**					
Anti-dsDNA	*131 (61.5%)*	*30 (69.7%)*	*101 (59..4%)*	**<0.001**	**<0.001**
Anti-dsDNA Titer 1:640 or higher	*49 (23.0%)*	*15 (34.9%)*	*34 (20.0%)*	**0.004**	**0.016**
Anti-Smith	*84 (39.4%)*	*16 (37.2%)*	*68 (40.0%)*	1.0	1.0
Low C3/C4	*101 (47.4%)*	*17 (39%)*	*84 (49.4%)*	0.146	0.233

^1.^ *p*-values were obtained utilizing the Wilcox Rank sum and Spearman’s rank correlation coefficient. FDR corrected *p*-values of ≤ 0.05 were considered to be statistically significant.

**Table 2:** Medication Use of Enrolled Subjects with Childhood-onset Systemic Lupus Erythematosus Stratified by Age ^2^.

Medication (at Enrollment)	Our Cohort (*n* = 213)	Early-Onset cSLE [Age at Onset *<*10] (*n* = 43)	Peri-Adolescent Onset cSLE [Age at Onset ≥10] (*n* = 170)	*p*-Value	Corrected *p*-Value
Prednisone	*151 (71%)*	*30 (69.7%)*	*121 (71%)*	0.842	0.982
Mycophenolate mofetil	*86 (40%)*	*14 (32%)*	*72 (42.3%)*	**0.003**	**0.0105**
Azathioprine	*17 (8%)*	*7 (12%)*	*10 (5.8%)*	**0.008**	**0.0186**
Hydroxychloroquine	*174 (82%)*	*30 (69.7%)*	*144 (84.7%)*	**<0.001**	**<0.001**
Cyclophosphamide	22 (*10%)*	*3 (6.9%)*	*19 (11.1%)*	0.579	0.982
Rituximab	*17 (8%)*	*1*	*16 (9.4%)*	0.994	0.994

^2.^ *p*-values were obtained utilizing Wilcox Rank sum and Spearman’s rank correlation coefficient. FDR corrected *p*-values of ≤0.05 were considered to be statistically significant.

## Data Availability

Data were ethically extracted from the patients’ file. Data used in this study are available from the corresponding author upon request.

## Supplementary Materials

The following supporting information can be downloaded at: https://www.mdpi.com/article/10.3390/children9121865/s1, Table S1: Neuropsychiatric and Dermatologic Characteristics of cSLE stratified by Age

## References

[1] Brunner H.I., Silverman E.D., To T., Bombardier C., Feldman B.M. (2002). Risk factors for damage in childhood-onset systemic lupus erythematosus: Cumulative disease activity and medication use predict disease damage. Arthritis Rheum..

[2] Bundhun P.K., Kumari A., Huang F. (2017). Differences in clinical features observed between childhood-onset versus adult-onset systemic lupus erythematosus: A systematic review and meta-analysis. Medicine.

[3] Ogbu E.A., Chandrakasan S., Rouster-Stevens K., Greenbaum L.A., Sanz I., Gillespie S.E., Marion C., Okeson K., Prahalad S. (2021). Impact of autoimmune cytopenias on severity of childhood-onset systemic lupus erythematosus: A single-center retrospective cohort study. Lupus.

[4] Tucker L.B., Uribe A.G., Fernandez M., Vila L.M., McGwin G., Apte M., Fessler B.J., Bastian H.M., Reveille J.D., Alarcón G.S. (2008). Adolescent onset of lupus results in more aggressive disease and worse outcomes: Results of a nested matched case-control study within LUMINA, a multi- ethnic US cohort (LUMINA LVII). Lupus.

[5] Pluchinotta F.R., Schiavo B., Vittadello F., Martini G., Perilongo G., Zulian F. (2007). Distinctive clinical features of pediatric systemic lupus erythematosus in three different age classes. Lupus.

[6] Tarr T., Dérfalvi B., Győri N., Szántó A., Siminszky Z., Malik A., Szabó A.J., Szegedi G., Zeher M. (2015). Similarities and differences between pediatric and adult patients with systemic lupus erythematosus. Lupus.

[7] Chow T.K., Looi L.M., Cheah P.L. (2015). A comparison of 1995 WHO classification with 2003 ISN/RPS classification of lupus nephritis: A single centre observation. Malays. J. Pathol..

[8] Abdwani R., Abdalla E., Al-Zakwani I. (2019). Unique Characteristics of Prepubertal Onset Systemic Lupus Erythematosus. Int. J. Pediatr..

[9] Massias J.S., Smith E.M.D., Al-Abadi E., Armon K., Bailey K., Ciurtin C., Davidson J., Gardner-Medwin J., Haslam K., Hawley D.P. (2020). Clinical and laboratory characteristics in juvenile-onset systemic lupus erythematosus across age groups. Lupus.

[10] Hui-Yuen J.S., Imundo L.F., Avitabile C., Kahn P.J., Eichenfield A.H., Levy D.M. (2011). Early versus later onset childhood-onset systemic lupus erythematosus: Clinical features, treatment and outcome. Lupus.

[11] Lehman T.J., McCurdy D.K., Bernstein B.H., King K.K., Hanson V. (1989). Systemic lupus erythematosus in the first decade of life. Pediatrics.

[12] Schuiling (2016). Women’s Gynecologic Health.

[13] Levy D.M., Kamphuis S. (2012). Systemic lupus erythematosus in children and adolescents. Pediatr. Clin. N. Am..

[14] Hochberg M.C. (1997). Updating the American College of Rheumatology revised criteria for the classification of systemic lupus erythematosus. Arthritis Rheum..

[15] Petri M., Orbai A.-M., Alarcón G.S., Gordon C., Merrill J.T., Fortin P.R., Bruce I.N., Isenberg D., Wallace D.J., Nived O. (2012). Derivation and validation of the Systemic Lupus International Collaborating Clinics classification criteria for systemic lupus erythematosus. Arthritis Rheum..

[16] Gladman D.D., Goldsmith C.H., Urowitz M.B., Bacon P., Fortin P., Ginzler E., Gordon C., Hanly J.G., Isenberg D., Petri M. (2000). The Systemic Lupus International Collaborating Clinics/American College of Rheumatology (SLICC/ACR) Damage Index for Systemic Lupus Erythematosus International Comparison. J. Rheumatol..

[17] (1999). The American College of Rheumatology nomenclature and case definitions for neuropsychiatric lupus syndromes. Arthritis Rheum..

[18] Moorthy L.N., Harrison M.J., Peterson M., Onel K.B., Lehman T.J. (2005). Relationship of quality of life and physical function measures with disease activity in children with systemic lupus erythematosus. Lupus.

[19] Alarcón G.S., McGwin G., Bartolucci A.A., Roseman J., Lisse J., Fessler B.J., Bastian H.M., Friedman A.W., Reveille J.D. (2001). Systemic lupus erythematosus in three ethnic groups. IX. Differences in damage accrual. Arthritis Rheum..

[20] Descloux E., Durieu I., Cochat P., Vital-Durand D., Ninet J., Fabien N., Cimaz R. (2009). Influence of age at disease onset in the outcome of paediatric systemic lupus erythematosus. Rheumatology.

[21] Danchenko N., Satia J.A., Anthony M.S. (2006). Epidemiology of systemic lupus erythematosus: A comparison of worldwide disease burden. Lupus.

[22] Johnson S.R., Urowitz M.B., Ibanez D., Gladman D.D. (2006). Ethnic variation in disease patterns and health outcomes in systemic lupus erythematosus. J. Rheumatol..

[23] Hiraki L.T., Silverman E.D. (2017). Genomics of Systemic Lupus Erythematosus: Insights Gained by Studying Monogenic Young-Onset Systemic Lupus Erythematosus. Rheum. Dis. Clin. N. Am..

[24] Lim S.S., Bayakly A.R., Helmick C.G., Gordon C., Easley K.A., Drenkard C. (2014). The incidence and prevalence of systemic lupus erythematosus, 2002–2004: The Georgia Lupus Registry. Arthritis Rheumatol..

[25] Koneru S., Kocharla L., Higgins G.C., Ware A., Passo M.H., Farhey Y.D., Mongey A.-B., Graham T.B., Houk J.L., Brunner H.I. (2008). Adherence to medications in systemic lupus erythematosus. J. Clin. Rheumatol..

[26] Popa-Lisseanu M.G., Greisinger A., Richardson M., O’Malley K.J., Janssen N.M., Marcus D.M., Tagore J., Suarez-Almazor M.E. (2005). Determinants of treatment adherence in ethnically diverse, eco- nomically disadvantaged patients with rheumatic disease. J. Rheumatol..

[27] Armstrong D.L., Reiff A., Myones B.L., Quismorio F.P., Klein-Gitelman M., McCurdy D., Wagner-Weiner L., Silverman E., Ojwang J.O., Kaufman K.M. (2009). Identification of new SLE-associated genes with a two-step Bayesian study design. Genes Immun..

[28] O’Neil K.M., Kickingbird L.M., Brunner H.I., Punaro M., Li S.C., Myones B.L., Zeft A.S. (2008). Clinical features of pre-pubertal onset systemic lupus erythematosus in girls. Arthritis Rheum..

[29] Webb R., Kelly J., Somers E., Hughes T., Kaufman K.M., Sanchez E., Nath S.K., Bruner G., Riquelme M.E.A., Gilkeson G.S. (2011). Early disease onset is predicted by a higher genetic risk for lupus and is associated with a more severe phenotype in lupus patients. Ann. Rheum. Dis..

