# Supplementary material for: Clinical Determinants of Childhood Onset Systemic Lupus Erythematosus among Early and Peri-Adolescent Age Groups
Source: Children (Basel). 2022 Nov 30;9(12):1865. doi: 10.3390/children9121865 (PMC9776488; doi:10.3390/children9121865)
Supplement: Supplementary file 1 [file children-09-01865-s001.zip › children-2006159-supplementary.pdf]

Table S1. Neuropsychiatric and Dermatologic Characteristics of cSLE stratified by Age

	Our cohort (*n* = 213)	Early-onset cSLE [Age at onset <10] (*n* = 43)	Peri-adolescent onset cSLE [Age at onset ≥10] (*n* = 170)
**Neuropsychiatric Dis-ease**			
Infarction	*4 (8.1%)*	0	*4 (2.3%)*
Neuropathy	*1*	*0*	*1*
Transverse Myelitis	*1*	*0*	*1*
Seizures	*2 (1%)*	*1*	*1*
Psychosis	1	*0*	*1*
Lupus Headache	*2 (1%)*	*0*	*2 (1%)*
**Rash**			
Vasculitic	*40 (18.7%)*	*12 (27.9%)*	*28 (16.5%)*
Malar	*66 (31.0%)*	*16 (37.2%)*	*50 (29.4%)*
Photosensitive	*52 (24.4%)*	*11 (25.6%)*	*41 (24.1%)*
Discoid	*5 (2.3%)*	*1*	*4 (2%)*

This table depicts the CNS and dermatologic manifestations of enrolled pediatric systemic lupus erythematosus.
